# Validation of the *Enterococci* indicator for bacteriological quality monitoring of beaches in Malaysia using a multivariate approach

**DOI:** 10.1186/2193-1801-2-425

**Published:** 2013-08-30

**Authors:** Asmat Ahmad, Ayokunle C Dada, Gires Usup, Lee Y Heng

**Affiliations:** School of Biosciences and Biotechnology, Faculty of Science & Technology, Universiti Kebangsaan Malaysia, 43600 UKM Bangi, Malaysia; Institute of Ecology and Environmental Studies, Obafemi Awlowo University, Ile-Ife, Nigeria; School of Environmental & Natural Resource Sciences, Faculty of Science & Technology, Universiti Kebangsaan Malaysia, 43600 UKM Bangi, Malaysia; School of Chemical Sciences and Food Technology, Faculty of Science & Technology, Universiti Kebangsaan Malaysia, 43600 UKM Bangi, Malaysia

**Keywords:** *Enterococci*, Indicator, Recreational beaches, Bacteriological quality monitoring, Multivariate approach, Malaysia

## Abstract

There is currently no established bacteriological beach quality monitoring (BQM) program in place in Malaysia. To initiate cost-effective, sustainable bacteriological BQM schemes for the ultimate goal of protecting public health, policy decision makers need to be provided robust, indigenous empirical findings that validate appropriate water quality parameters for inclusion in such monitoring programs. This is the first study that assesses the validity of *enterococci* as an ideal indicator for bacteriological BQM in Malaysia using a multivariate approach. Beach water and sand samples from 7 beach locations were analyzed for a total of twenty-one microbial and non-microbial water quality parameters. A multivariate approach incorporating cluster analyses (CA), principal component analyses (PCA), and factor analysis (FA) was also adopted. Apart from the weak correlations of *Staphylococcus aureus* with concentrations of *Vibro* species (r = 0.302, p = 0.037) and total coliforms (r = 0.392, p = 0.006) in seawater, no correlation existed between *S. aureus* concentration and other parameters. Faecal coliforms failed to correlate with any of the tested parameters. *Enterococci* also correlated with more quality parameters than faecal coliforms or any other indicator. Multiple linear regressions highlighted a significant, best fit model that could predict *enterococci* concentrations in relation to other parameters with a maximum predictive success of 69.64%. PCA/FA clearly delineated *enterococci* and faecal coliforms as parameters that weighed strongly for BQM while *Staphylococcus aureus*, faecal coliforms and *enterococci* weighed strongly for beach sand quality monitoring. On the whole, higher correlations of *enterococci* levels with other parameters than was observed for faecal coliforms suggest that the former be considered a preferred parameter of choice for BQM in Malaysia. Our findings provide meaningful evidence particularly as it relates to the correlation of *Enterococci* with pathogens and other non-microbial parameters. It also provides empirical data to validate the applicability of the *enterococci* indicator paradigm for bacteriological beach quality monitoring in Malaysia. The current study thus provides policy decision makers evidenced based approach to parameter streamlining for optimized beach sampling and sustainable bacteriological quality monitoring.

## Background

In Western nations, beach water and sand quality monitoring has attracted significant attention in recent years owing to improved legislation (Casas et al., 2011). This development partly may be due to the availability of institutional frameworks-scientific, managerial and engineering competencies, considerable budgetary allocations and relevant political and stakeholder willpower that concertedly work to ensure the functionality of robust surveillance systems. Yet in these countries, there have been accounts reported of alarming levels of bacteria found in faeces and dangerous heavy metals in contaminated storm water flowing into beaches (Oshiro and Fujioka, 1995;Olapade et al., 2006;Grant et al., 2001;Novais et al., 2005;Yoder et al., 2004;Debacker et al., 2000). This often results in a number of public health advisories and beach closures (Graham et al., 2009;Casas et al., 2011). Furthermore, epidemiological research on the effects on health of swimming at bathing beaches has shown that swimming in bathing beaches carries some risk of illness even when these beaches comply with existing legislative standards (Barrell et al. 2000).

The situation may be particularly worrisome in less economically developed countries where legislative standards do not exist as a result of frail institutions and tight budgets. In such cases, a prevailing absence of surveillance schemes may allow in beaches undetected pollution from stormwater, domestic sewage and industrial effluents (Kuylenstierna et al., 2009). The situation is exacerbated as less developed nations are often at a loss on how to develop workable standards for the purpose of beach water quality monitoring (Johnstone, 2007). Reimann and David Banks (2004) also highlighted the problems of setting meaningful action levels or maximum admissible concentrations (MAC-values) for water quality. Usually, public authorities and responsible politicians consider water quality statistics and take a decision on what proportion of failures they think can be accepted based on a consideration of political factors, economic factors and suggested MAC hinged on available empirical data. In cases where there are no indigenous studies that present empirical findings which suggest epidemiologically proven action levels or maximum admissible concentrations that make a case for the initiation of a beach water bacteriological quality monitoring (BQM) program, a worst case scenario of political inaction and negligence may become observable (Dada et al., 2012).

Although, with a more or less generalist approach, a surveillance program is currently in place for marine water quality (DOE, 2006) that lays emphasis on levels of E.coli, oil and grease, total suspended solids and selected heavy metals. Public recreational beaches are however, apparently left out of these surveillance schemes. A recent report highlighted challenges in the management of coastal recreational beaches in Malaysia (Dada et al., 2012). Particularly worrying is the possibility of direct sewage and stormwater discharge into beaches coupled with the apparent absence of early warning systems that inform the public on how safe the beaches are. Also recent review articles on recreational beaches in Malaysia have highlighted potential impacts of tourism activities, shipping, refinery effluent, land reclamation and coastal zone property development on recreational water quality (Dada et al., 2012;Praveena et al., 2011). Every year, an estimated 120 million cases of gastrointestinal illnesses and 50 million cases of respiratory ailments are contracted by bathers in polluted coastal waters (Shuval, 2003). It could thus be posited from a public health point of view, that there is the need for the development of a specific bathing beach water bacteriological quality monitoring (BQM) scheme in Malaysia.

Agreeably, these bathing beach bacteriological quality monitoring schemes will be built on data generated from for a dependable information collection system capturing output from indigenous studies with carefully planned experimental designs that aim to support early warning systems, trigger public behavioural change and the political will power needed for high-level policy decision making. The challenge at hand is thus no longer that of generating or accumulating beach water quality data but one of integrating information in a systematic manner for the purpose of making decisive policy judgements on beach water quality management (Dada et al., 2012). Given the intricacies associated with study design, methodology, data collation and interpretation in water quality monitoring schemes (Ongley, 1997), it is imperative that a robust approach be adopted for interpretation of generated data so that the original purpose is not defeated.

One of such approaches is the use of multivariate analysis for data extrapolation. A number of multivariate statistical techniques, such as cluster analysis (CA), principal component analysis (PCA), factor analysis (FA) and discriminant analysis (DA) offer significant assistance in the interpretation of complex data matrices. The outcome is thus a generally improved understanding of the water quality and ecological status of the studied systems. The application of multivariate statistical techniques also enhances the identification of possible factors that influence water systems. These undoubtedly offer a valuable tool for reliable and sustainable management of water resources (Lee et al., 2001;Adams et al., 2001;Alberto et al., 2001;Reghunath et al., 2002;Simeonov et al., 2003,2004). Previously published literature indicate that multivariate statistical techniques have successfully assisted in characterizing surface and freshwater quality (Helena et al., 2000;Singh et al., 2004,2005). For example, multivariate statistical methods in a regional study that predicted non-point heavy metals sources in soil (Facchinelli et al., 2001). Multivariate approach also helped in interpreting a large chunk of complex data matrix produced after an evaluation of several surface water parameters in Northern Greece (Simeonov et al., 2003). Another principal application of multivariate statistical analysis particularly for the identification of contributory pollutant sources was explored in another study by Terrado et al. (2006). Sherestha and Kazama (2007) also adopted CA, PCA, PFA, and discriminant analysis as multivariate techniques in the evaluation of temporal and spatial variations of a large complex water quality data set generated from their study on the Fuji River basin.

 (USEPA Going beyond mere making a case for the birth of a specific BQM scheme in the Malaysian context, there is the need to evaluate various water quality parameters for appropriateness and inclusion in the envisioned BQM scheme. This will reduce labour and ensure that money is not wasted on inappropriate or redundant parameters. To evaluate beach water quality, various microbial groups have been used as indictors of contamination. These include the *E. coli, Enterococcus*^2004^;Sinigalliano et al., 2010), *C. perfringens* (Wiedenmann et al., 2006;Miller et al., 2010), and *Staphylococcus aureus* (Abdelzaher et al., 2010). As this group of indicator organisms was selected based on epidemiological studies carried out mostly in temperate waters of the developed world (WHO, 2003), it has been questioned whether these could be applicable to resort beaches in tropical or subtropical climates (Curiel-Ayala et al. 2012). Tenets of this school of thought have thus proposed the introduction of other indicator organisms for the evaluation of recreational waters, in the conditions which obtain in subtropical settings (Salas and Robinson, 2006) with typical day temperature ranging from 24°C to 29°C. In hotter tropical climates like Malaysia, where temperatures could reach as high as 35°C, the situation may apparently not be the same bearing in mind the effect of temperature on microbial growth dynamics. Arriving at a choice parameter for beach water quality monitoring may thus warrant the need for indigenous studies that comparatively present assessments of these microorganisms used as indictors of beach water pollution.

To enhance the birth of a cost-effective, efficient BQM scheme for the ultimate goal of sustainable beach management and public health protection in Malaysia, the current study thus aims at providing empirical findings that validate an appropriate water quality parameter for adoption in BQM. This is the first study that assesses the validity of *enterococci* as an ideal indicator for BQM in Malaysia using a multivariate approach. Partly, we aimed at generating spatial data related to quality of these beaches. Secondly, we aimed at generating correlations between *Enterococci* and other indicator organisms, between *Enterococci* and other water quality parameters used in water quality monitoring. Our third objective was to explore the use of multivariate techniques in validating an *Enterococci-*based indicator organism paradigm applicable for beach water bacteriological quality monitoring in Malaysia.

## Results and discussion

### Physicochemical parameters

The physicochemical parameters in seawater and sediments collected from all location were determined and presented in Table 1. The temperature of all water samples tested ranged from 26.6 to 29.56 ± 0.8°C. Conductivity ranged between 0.08 and 28.70 ± 0.05 and dissolved oxygen between 1.99 ±0.18 and 7.60 ± 0.12. Recorded pH values (7.00-8.36) differed depending on the sample location.

**Table 1:** **Physico-chemical parameters**

Beach	T (W)	D (W)	S (W)	DO (W)	pH (W)
SRI 7 1A	28.23 ± 0.01	26.74 ± 2.82	16.85 ± 0.92	7.10 ± 0.10	8.14 ± 0.03
SRI 7 1B	28.85 ± 0.01	2.75 ± 0.00	1.41 ± 0.00	5.94 ± 0.02	7.88 ± 0.02
SRI 72A	28.47 ± 0.01	27.30 ± 0.20	17.02 ± 0.13	6.96 ± 0.24	8.24 ± 0.01
SRI 73	28.15 ± 0.00	1.96 ± 0.03	0.99 ± 0.02	1.99 ± 0.18	7.44 ± 0.05
PCB 1A	28.23 ± 0.04	24.22 ± 3.63	12.52 ± 0.46	7.60 ± 0.12	8.24 ± 0.02
PCB 1B	28.28 ± 0.01	26.82 ± 1.14	17.73 ± 0.00	7.51 ± 0.08	8.23 ± 0.00
TOK BALI 1A	29.20 ± 0.01	3.32 ± 0.01	1.73 ± 0.01	4.52 ± 0.06	7.00 ± 0.03
TOK BALI 1B	29.05 ± 0.01	22.85 ± 0.43	13.91 ± 0.12	7.56 ± 0.03	7.84 ± 0.04
KUALA BESAR 1A	26.60 ± 0.00	0.17 ± 0.00	0.08 ± 0.00	2.02 ± 0.27	6.80 ± 0.01
KUALA BESAR 1B	27.15 ± 0.04	17.39 ± 1.67	8.98 ± 0.46	7.81 ± 0.02	7.95 ± 0.06
PANTAI SENUK 1A	28.63 ± 0.03	26.48 ± 0.50	18.20 ± 0.02	7.15 ± 0.02	8.31 ± 0.00
PANTAI SENUK 1B	28.45 ± 0.01	1.77 ± 0.03	0.89 ± 0.01	3.92 ± 0.08	7.54 ± 0.01
PAITAI IRAMA 1A	28.78 ± 0.66	44.70 ± 0.44	28.70 ± 0.05	6.91 ± 0.06	8.30 ± 0.01
PANTAI IRAMA 1B	29.56 ± 0.08	42.88 ± 1.10	27.66 ± 0.65	6.92 ± 0.06	8.34 ± 0.01
PANTAI SABAH	28.66 ± 0.01	34.03 ± 0.15	20.21 ± 0.94	7.36 ± 0.17	8.37 ± 0.01

### Population density and patterns

All values of population counts were log transformed prior to statistical analysis. Geometric means were thus recorded after the analysis as mean log values ± standard deviation. The mean total plate count observed for all studied beach water samples ranged from 6.01 ± 0.02 log CFU/100 ml to 9.89 ± 0.01 logCFU/100 ml. The highest and lowest counts were observed for samples collected from Tok Bali 1B (9.89 logCFU/100 ml) followed closely by Paintai Irama (9.86 logCFU/100 ml), Kuala Besar 1B (9.73 logCFU/100 ml) and Pantai Sabak (9.68 logCFU/100 ml). Pantai Sri Tujuh 1A & 1B, PCB1A and Pantai Senuk 1B ranked lowest in the mean log aerobic bacteria counts (6.01 ± 0.02 CFU/100 ml). For the beach sand samples, generally higher counts were observed, with the mean log aerobic counts ranging from 10.23 ± 0.00 to 13.53 ± 0.02 CFU/100 ml. The lowest mean log counts of aerobic bacteria observed for sand samples was observed for beach sediment collected from Sri Tujuh 2B (10.22 ± 0.01) and PCB 1B beaches (10.25 ± 0.01).

For *Enterococci* counts in beach water samples, mean log counts ranged from 0 to 2.55 ± 0.06 CFU/100 ml. Notably, *Enterococci* was not detected in samples from two beaches, Pantai Irama IA and Pantai Irama IB. Apart from these two beaches, *Enterococci* counts were comparatively lowest in Paintai Sri Tujuh 1A and Paintai Sri Tujuh 1B water samples. Analysis of recorded mean log counts for *Enterococci* in beach sand samples revealed more beaches with no detection of *Enterococci* as compared with water samples as Paintai Sri Tujuh 1A, PCB 1A, Tok Bali 1A, Tok Bali 1B, Kualar Bbesar 1B, Paintai Irama 1A and 1B all yielded negative results during the enumeration of *Enterococci* in the collected beach sediments. Higher mean log counts however were observed for sand samples collected from Paintai Sri Tujuh 3 beach (1.31 ± 0.01 logCFU/100 ml). In a few instances, higher *Enterococci* counts in beach water was recorded than in beach sand particularly for Pantai Sri Tujuh 1B and Pantai Sri Tujuh 3. Notably, most beach sand samples commonly harboured fewer *Enterococci* than the beach water samples. This difference however was not statistically significant as p was found greater than 0.99 for the mean logCFU/100 ml comparisons. Currently, there is no enforceable standard available in Malaysia for designated bathing beaches to comply with. Also no *enterococci* standards exist in Malaysia for the purpose of comparison. Comparing *enterococci* levels in this study with the tabulated benchmark documented for bathing waters in Barrell et al. (2000) in concert with the Bathing Waters Regulation (1991), counts obtained in this study was in one of the beaches up to two-fold higher than the recommended guide levels. Usually the risk to health increases in proportion to the amount of faecal pollution as measured by indicator organisms.

Using a baseline approach for all sampled beaches involving the direct use of a detection limit of one colony forming unit per 100 μl aliquot of water samples, only four beaches were found to contain elevated levels of *Salmonella typhi* to be detectable at this limit with mean log counts ranging from a transformed value of 3.03 ± 0.46 cfu/100 ml to 4.70 ± 0.01 CFU/100 ml. *Vibrio cholerae* was not detectable in six out of the sixteen beach locations considered in this study. In beach water samples where it was detected, it was detected at a mean log count range of 3.00 ± 0.00 to 6.33 ± 1.15 cfu/100 ml. For beach sand analysis, while most sediment samples contained fewer levels of Vibrio cholera than their water sample counterparts, counts obtained for *Vibrio* ranged from 3.30 ± 0.01 to 6.90 ± 1.53 logCFU/100 ml. Counts were recorded for other non-*cholerae Vibrio* species in both beach water ad sediment samples. For beach water samples, apart from samples collected from Pantai Sri Tujuh 1B, 2A, 3 and Kuala Besar 1B, tested water samples were found negative. Positive samples for *Vibrio* spp. were found to have counts that ranged from 3.48 ± 0.01 logCFU/100 ml (Sri 72B) to 6.33 ± 1.53 logCFU/100 ml (PCB 1B). Of all the sand samples analysed, only six sampled beaches were found positive to *Vibro* species with a mean log counts of 3.301 ± 0.01 CFU/100 ml (Pantai Irama1A, Pantai Sri Tujuh 2B), 4.18 ± 0.01 CFU/100 ml (Pantai Senuk), 4.43 ± 0.98 CFU/100 ml (Pantai Sri Tujuh 2A), 6.05 ± 1.65 CFU/100 ml (Pantai Sri Tujuh 3) and 6.43 ± 1.47 CFU/100 ml (Pantai Senuk).

Values obtained for mean log of total coliform counts for beach water samples ranged from 3.0 ± 0.01 CFU/100 ml (PCB1A) to 6.67 ± 1.68 CFU/100 ml (Kuala Besar 1A). Total coliform counts for water samples were numerically higher among beach sand samples although the difference was found not to be statistically significant as p value obtained from the analysis of variance of means was greater than 0.99. Faecal coliforms were only detectable at Kuala Besar 1B beach water samples but not in the beach sand samples. Similarly only in two beaches (Pantai Sri Tujuh 3 and Tok Bali 1A) were faecal coliforms detected with mean log counts higher than 4.0 per 100 ml of seawater sample. Faecal coliforms are regarded as more specific indicators of faecal contamination than total coliforms. It is interesting to note that comparing data obtained in this study with the Bathing Waters Regulation (1991), a guide and imperative level of 100 cfu/100 ml and 2000/100 ml (equivalent to 1.0 logcfu/ml and 3.30 logcfu/ml respectively), faecal coliform counts considerably complied with this standard. Only one of the beaches failed to comply and unlike the case of *enterococci* count (WHO, which appeared to be more sensitive capturing another two beaches that failed to comply to the stipulated standards. Our observation is in concert with studies that have shown that the bacterial indicator most strongly associated with risk to health seems to be the *enterococci*^2003^;Fleisher et al., 1993).

### Pictorial relationships between *Enterococci* and other parameters measured in the study

Patterns observed in the readings of physico-chemical parameters and concentrations of microbial parametars were observed using three dimensional surface plots that show pictorial inter-relationships between the tested parameters. The parameters used in the plots are (1) TPCS-total heterotrophic plate count of beach sediment samples (2) EW- *Enterococci* concentration in seawater (3) ES- *Enterococci* concentration in beach sediment samples (4) SW- *Salmonella* typhi concentration in beach water samples (5) SAW- *Staphylococcus aureus* concentration in beach water samples (6) VCW- *Vibro cholerae* concentration in beach water samples (7) VCS- *Vibrio cholerae* concentration in beach sediment samples (8) VSW- *Vibrio* spp. concentration in beach water samples (9) VSS – *Vibrio* species concentration in beach sediment samples (10) TCW – Total coliform count of beach water samples (11) TCS- Total coliform count of beach sediment samples (12) FCW – Faecal coliform count of beach water samples (13) FCS- Faecal coliform count of beach sand samples (14) AW- *Aeromonas hydrophila* concentration in beach water samples (15) EW- *Enterococci* concentration in beach water samples (16) SAS- *Staphylococcus aureus* counts of beach sediment samples (17) T- Beach water temperature in °C (18) D - Dissolved oxygen in % air saturation (19) DO - Dissolved oxygen in mg/L (20) pH- measure of the activity of the hydrogen ion concentration of beach water sample (21) S- Salinity (in ppt). Variables (1) – (16) were recorded in logCFU/100 ml. Data was pooled from all the beaches for the analyses.

**Figure 1:**
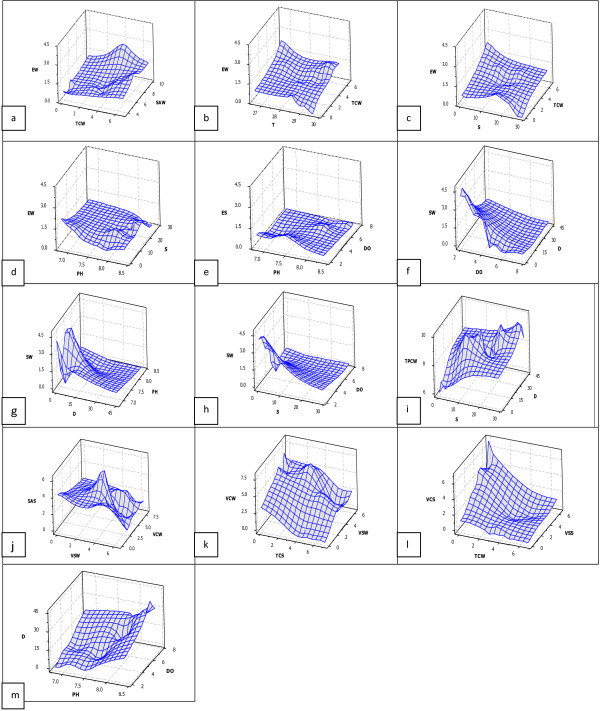
**Three dimensional surface plots depicting pictorial representation of relationships between selected parameters, a-m being plots of EW/TCW/SAW, EW/T/TCW, EW/S/TCW, EW/pH/S, ES/pH/DO, SW/DO/D, SW//D/pH, SW/S/DO, TCPS/S/D, SAS/VSW/VCW, VCW/TCS/VSW, VCS/TCW/VSS and D/pH/DO respectively.** (1) TPCS-total heterotrophic plate count of beach sediment samples (2) EW- *Enterococci* concentration in seawater (3) ES- *Enterococci* concentration in beach sediment samples (4) SW- *Salmonella* typhi concentration in beach water samples (5) SAW- *Staphylococcus aureus* concentration in beach water samples (6) VCW- *Vibro cholerae* concentration in beach water samples (7) VCS- *Vibrio cholerae* concentration in beach sediment samples (8) VSW- *Vibrio* spp. concentration in beach water samples (9) VSS – *Vibrio* species concentration in beach sediment samples (10) TCW – Total coliform count of beach water samples (11) TCS- Total coliform count of beach sediment samples (12) FCW – Faecal coliform count of beach water samples (13) FCS- Faecal coliform count of beach sand samples (14) EW- *Enterococci* concentration in beach water samples (15) T- Beach water temperature in °C (16) DO - Dissolved oxygen in mg/L (17) pH- measure of the activity of the hydrogen ion concentration of beach water sample (18) S- Salinity (in ppt).

Two major sets of patterns are presented (i) the ones showing how other parameters (microbial and non-microbial) vary with each other (Figure 1f - 1m) (ii) patterns showing how *Enterococci* varies with other parameters (Figure 1a - 32). From the surface plots generated, increasing concentrations of total coliform in beach water samples corresponded with increasing concentrations of *Enterococci*. A similar observation was observed between the concentrations of *Enterococci* and *Staphylococcus aureus* in water (2a). *Enterococci* concentration remained fairly constant at temperatures between 26-28°C but gradually declined at temperatures above 28°C (2b). *Enterococci* concentration in seawater gradually increased with increasing salinity levels but began a steady decline at salinity levels above 15 ppt (2c). *Enterococci* concentration in seawater generally reduced with increasing pH values above neutral (2d). However, the case for *Enterococci* concentration in beach sand was somewhat different as concentration of *Enterococci* reduced as pH levels dropped below 7 and picked up at neutral ph value before gradually declining as pH advanced into more alkaline values (2e). Generally, *Salmonella typhi* count in water (SW) tended to decrease with increasing DO (2f). At increasing pH values however, SW decreased with increasing D value (2g). Again, *Salmonella typhi* count in water tends to decrease with increasing salinity (S) (2h). Total plate count of bacteria in seawater generally increased with increasing salinity (S) (2i). *Staphylococcus aureus* concentration in sand reduced with increasing concentration of *Vibro* species in seawater albeit in a fluctuating manner (2j). *Vibrio cholerae* concentration in seawater generally reduced with increasing levels of TCS (3k) and FCW (2m) and with reducing levels of *Vibrio* species (2n) while *Vibro cholerae* concentration in beach sand reduced with reducing levels of *Vibrio* species in beach sand (2l). Generally, dissolved oxygen concentration increased with increasing pH (2m).

It should however be noted that these pictorial representation from the data obtained are just raw presentations of how the parameters vary with one another. In statistical terms, a more reliable approach is to use correlation analysis that presents information on how each parameter correlates with the others. The major consideration besides the correlation ranking here is the significance levels. Using a Pearson correlation analysis, it was observed that *Enterococci* concentrations significantly correlated to more number of parameters than any other microbial or non-microbial parameter analyzed in this study (additional data). Both seawater and sand concentrations of *Enterococci* correlated in varying strengths to the concentration of *Salmonella typhi* in water (r = 0.445, p = 0.002 and r = 0.739, p = 0.000 respectively). The correlation between *Enterococci* concentration in sand and the concentration of pathogens in seawater as was observed in our study supports the position of other studies that suggested beach sand can be a source of faecal indicator bacteria and pathogens to adjacent waters (Oshiro & Fujioka, 1995;Oliveira & Mendes, 1992).

The correlation of *Enterococci* in seawater however was inversely correlated to temperature (r = −0.590, p = 0.000), salinity (r = −0.386, p = 0.009) and dissolved oxygen (r = −0.537, p = 0.000). The negative correlation of water temperature to *Enterococci* antogonised the concerns that indicator organisms may grow in the environments as argued by previous studies (Beversdorf et al., 2007;Shah et al., 2011;Phillips et al., 2011,2012). It may be that the organism is not significantly viable at this temperature taking into account the prevailing pH, salinity, oxygen content and other physicochemical parameters of the beach water samples tested in our study. Hopefully studies will emerge in the future that substantiate this observation while specifically exploring the survival potential of *Enterococci* in bathing beach water and beach sand in the tropical context available in Malaysia. In our study, *Enterococci* concentration in beach sand correlated inversely but in stronger terms with dissolved oxygen levels in seawater. Apart from the weak correlation of *Staphylococcus aureus* concentration in seawater with *Vibro* species (r = 0.302, p = 0.037) and total coliforms (r = 0.392, p = 0.006) in seawater, no other correlations existed between *Staphylococcus* concentration and other parameters. Similarly, concentration of *Staphylococcus aureus* in beach sand correlated weakly with those of *Vibro* species (r = 0.360, p = 0.012) and total coliforms (r = 0.595, p = 0.000) in beach sand. This observation questions the adoptability of *S. aureus* for use as beach water quality indicator in the considered location. Our findings however contradict recent comparative study by Goodwin et al. (2012) that showed strong correlations in seawater and sand between water temperature and seawater concentration of *Enterococci*. The reason for this might be because the study was conducted in possibly temperate bathing waters in the United States. Our study was conducted in a tropical setting with typically high day temperature reaching up to 33°C. This might have significant implications on population dynamics of the microorganisms studied. Notwithstanding, our findings are in concert with others that reported that *S. aureus* do not correlate with various other indicators (El-Shenawy, 2005;Calderon et al. 1991;Selvakumar and Borst, 2006;Yamahara et al., 2012;Enns et al., 2012).

Notably, faecal coliform concentration in seawater did not correlate with any of the parameters tested apart for inverse correlations with *Vibro cholerae* (r = −0.436, p = 0.02) in seawater. This observation presents a shadow of doubt on the appropriateness of faecal coliform concentration for seawater quality monitoring in the studied location. Interestingly also, faecal coliform concentration in sand also did not correlate with any of the tested beach sand quality parameters. On a general note, the results of the correlation analysis apparently indicate that *Enterococci* concentrations seem to be a preferred choice indicator bearing in mind that it correlated more and better with other microbial parameters. This observation is in concert with previous studies (WHO, 2003;Fleisher et al., 1993).

### Regression analysis

**Figure 2:**
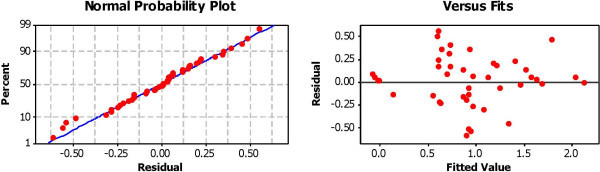
**Residual plots for generated*Enterococci*model validation.**

Statistical Relationships between *enterococci*, other microbial and non-microbial parameters were explored using a combination of simple and multiple linear regression analysis. First, *enterococci* count (in logcfu/ml) was compared with other microbial indicators tested for water and sand respectively. Again *enterococci* counts of water samples were compared with varying combinations of the other parameters analyzed in this study. The results of regression analysis are presented in Table 2. Each of the parameters were found to have a extremely significant relationship with *Enterococci* count (p < 0.0001). A number of studies have employed multivariate linear or non-linear approaches for studying relationships between varying environmental parameters. Regardless of the model, r^2^ value is generally accepted as a standard criterion for the predictive success of models (Håkanson et al., 2003). However, it should be noted that in some instances, a univariate simple regression model could yield high r^2^ values but may fail to define the complex relationship between water quality parameters. This has prompted for the need to employ multivariate techniques. Nonetheless, determination coefficient (r^2^) remains a popular tool in environmental modeling. To ensure that inherent errors associated with this approach was not the case for the model generated, the model validity was evaluated as described by MINITAB using error terms (or residuals) that have a mean of zero and constant variance. To check these assumptions, residuals versus fitted values plot and normality probability plots of the residuals were generated (Figure 2) and checked for constant variance while confirming that the residuals are scattered randomly around zero. The simple regression model generated in this study was able to predict with a maximum predictive success of 69.64% using just a combination of 5 parameters. The model used to predict *Entercocci* counts in water samples is given as:1

**Table 2:** **AVOVA results of regression analysis (n = 48)**

Included independent variables	Regression coefficient (bk)	Standard error of bk	T	P	R^2^(%)
Constant	16.5116	2.65543	6.21805	0.000*	69.64
TPCS	0.1992	0.06151	3.23898	0.002*	
SAW	0.1298	0.03961	3.27766	0.002*	
VCW	−0.0589	0.01755	−3.35683	0.002*	
AW	−0.0893	0.02165	−4.12448	0.000*	
DO	1.8938	0.55339	3.4221	0.002*	
pH	−21.8692	3.48283	−6.27915	0.000*	

*p <0.01, TPCS-total heterotrophic plate count of beach sediment samples, SAW- *Staphylococcus aureus* concentration in beach water samples, VCW- *Vibro cholerae* concentration in beach water samples, VCS- *Vibrio cholerae* concentration in beach sediment samples, AW- *Aeromonas hydrophila* concentration in beach water samples, EW- *Enterococci* concentration in beach water samples, DO - Dissolved oxygen in mg/L (20) pH- measure of the activity of the hydrogen ion concentration of beach water sample.

Where EW is the beach water concentration of *Enterococci* in logCFU/100 mL, TPCS is the total heterotrophic plate count (in logCFU/100 mL) of soil samples collected beneath the surfing zone where water samples were collected, SAW is *Staphylococcus aureus* concentration of beach water samples (in logCFU/100 mL), VCW is *Vibrio cholerae* concentration of beach water sample (in logCFU/100 mL), AW is the *Aeromonas hydrophila* concentration (in logCFU/100 mL), DO is dissolved oxygen in water (in mg/L) and pH (W) being the pH value of beach water sample. It was interesting to note that our analysis produced a model which could factor in *S. aureus* concentration in its prediction of *enterococci* concentrations in seawater. Goodwin et al. (2012) also arrived at a best-fit model that related *S. aureus* in seawater with *enterococci* levels. The predictive model that could predict *enterococci* levels in beach water based on *Staphylococcus aureus* counts alone in water, although significant, had a low predictive power of 27.03%. Notably, when SW, TCS and TCW were each added to the model, the predictive power of the generated model increased to 70.10, 70.89 and 74.52% respectively. However, these were not statistically significant we attempted a predictive model that could predict *Enterococci* counts based on the levels of other conventional bacterial indictors for water quality. When only either of total coliforms and faecal coliforms were incorporated, the predictive ability of the obtained model were very low, 29.20% and 40.36% respectively for the model EW = 0.612 + 0.169TC and EC = 0.6263 - 0.02704FCW + 0.16771TC.

### Spatial similarity, site groupings and variations in beach water quality

**Figure 3:**
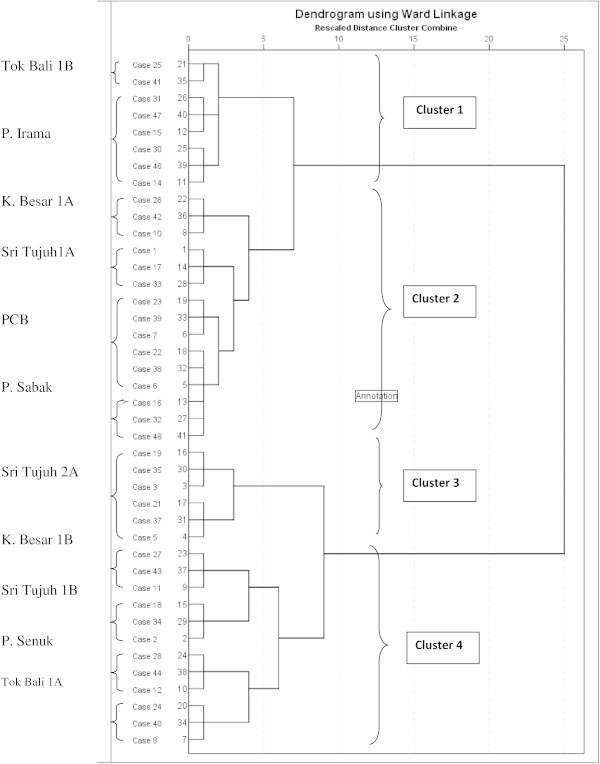
**Generated dendogram clustering sampling locations based on water quality.**

Cluster analysis was applied to distinct spatial similarity between the selected sampling sites. The obtained dendogram presented in Figure 3 shows groupings of all the beach sampling sites into four statistically significant clusters. As specific information documented on these beaches particularly the potential point sources of pollution are not available, visual observation of these beaches were used as a basis for classification in a bid to compare with cluster analysis. The clusters obtained from this analysis are also in agreement with these considerations. In this study, clusters 1 to 4 correspond to increasing levels of pollution in that order. We applied an approach that fed into the data matrix used for cluster analysis separate data for each of the sampling sites per beach. This was necessary to allow a reflection of specific polluted sections of each considered beach as opposed to the use of pooled data. For instance, in a separate study on Teluk chempedak, the portion of the beach receiving direct river water discharge had higher levels of pollution as compared to another site on the same beach not receiving river water discharge.

A similar observation was noted at Teluk Kemang in a separate study where a portion of the beach in the main bathing area receiving direct storm water discharge had significantly higher loads of antibiotic resistant bacteria as compared to other bathing sites not directly receiving pollution loadings from the stormwater discharge pipes. Obviously from our clustering results, three of the beaches (Tok Bali, Sri 7 and Kualar Besar) had sampling sites grouped in more than one cluster as opposed to a situation where all sampling sites on the same beach are clustered in a group. This observation supports the dynamic nature of microbial loadings in beaches. It should thus be noted for studies that will emerge in the future, sampling spots that do not representatively cover the entire length of any beach in question may be far from presenting an accurate picture of the overall quality of the beach. It is also recommended that future strategies for BQM in Malaysia should include a zoning strategy for individual beaches such that each zone of a beach in question is classified based on the potential pollution input and associated level of threat or risk to public health. With this system in place, bathers are able to make an informed choice on which portion of the beach to bath. Although factors that drive bathers preference for bathing sites within a beach is albeit complex and subjective, from physical observation of beaches in Malaysia, it could be noted that bathers on arriving at a designated beach generally would make a choice on which portion of the beach to bathe based on nearness to amenities such as food stalls, relaxation tents, speed boats etc. In a previous study, the section of Teluk Kemang beach with intense recreational activity is also very close to a storm water discharge pipe. Yet there are no early warning systems to emphasize to the public, the need to avoid swimming in the vicinity of storm water drains during or a few days after rainfall events as these drains are potential sources of pollution.

Another approach that could be built based on results of clustering analysis of data obtained from BQM is beach profiling. Unlike the beach zoning previously iterated involving multiple zones per beach, beach water profiling is a general picture involving a classification system for several beaches, in which case, multiple beaches per class or zone. Beach water profiling is increasingly gaining increasing acceptance in some countries. A fundamental objective of this approach is to allow the citizenry to make an informed choice on which beach to choose for recreational activities. This is done via the annual public disclosure in the form of a report, the bacteriological quality of available coastal and freshwater bathing waters. Clusters built from data generated from BQM could be aligned with other factors to produce a profiling system that includes a general description of the bathing water, where wastewater outfalls and combined sewer overflows (CSOs) are positioned, potential sources of pollution, management measures, the location of the sampling points and how the beach has performed against bathing quality ratings over the previous months and years. With a beach profiling program in place, beaches could then be classified as good, fair or poor based on pre-defined quality criteria counts. It is noteworthy to assert that relevant agencies in Malaysia have made giant strides in this direction particularly for rivers in Malaysia. Water quality data from the 143 river basins throughout Malaysia are used to determine the water quality status weather in clean, slightly polluted or polluted category and to classify the rivers in Class I, II, III, IV or V based on Water Quality Index (WQI) and Interim National Water Quality Standards (INWQS) for Malaysia every year (Dada et al 2012). It is hoped that a similar classification scheme be developed for recreational beach waters. A further improvement of this approach will involve the designation of beach managers for each beach who will be responsible for putting up signs at each designated bathing waters advising on the beach class, current beach water quality and potential pollution sources.

### Compositional patters of quality and underlying factors

**Figure 4:**
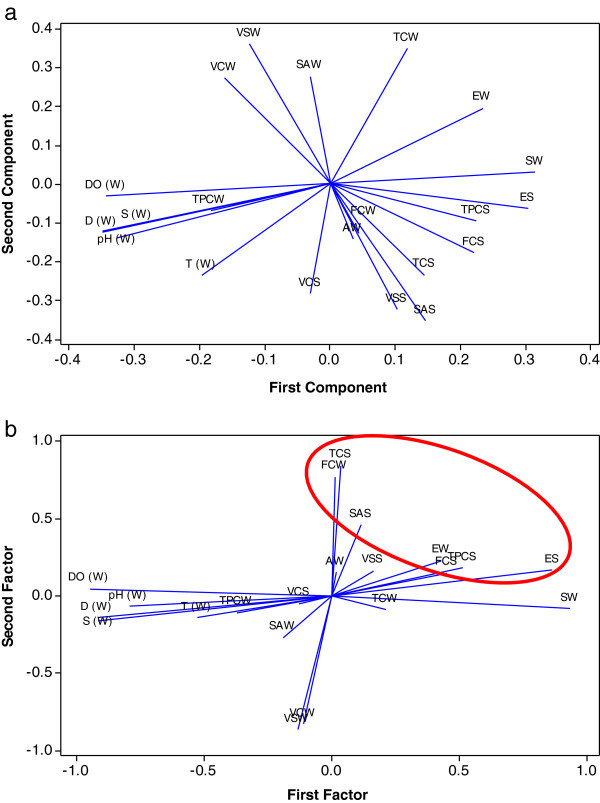
**(a): Loading plots based on contribution of each quality parameter (a) towards the first and second component in PCA and (b) towards the first and second factor in FA (1) TPCS-total heterotrophic plate count of beach sediment samples (2) EW-*Enterococci*concentration in seawater (3) ES-*Enterococc*concentration in beach sediment samples 4) SW-*Salmonella*typhi concentration in beach water samples (5) SAW-*Staphylococcus aureus*concentration in beach water samples (6) VCW-*Vibro cholerae*concentration in beach water samples (7) VCS-*Vibrio cholerae*concentration in beach sediment samples (8) VSW-*Vibrio*spp concentration in beach water samples 9) VSS –*Vibrio*species concentration in beach sediment samples (10) TCW – Total coliform count of beach water samples 11) TCS- Total coliform count of beach sediment samples (12) FCW – Faecal coliform count of beach water samples (13) FCS- Faecal coliform count of beach sand samples (14) AW-*Aeromonas hydrophila*concentration in beach water samples (15) EW-*Enterococci*concentration in beach water samples (16) SAS-*Staphylococcus aureus*counts of beach sediment samples (17) T(W)- Beach water temperature in 0C (18) D(W) - Dissolved oxygen in % air saturation (19) DO(W) - Dissolved oxygen in mg/L (20) pH(W)- measure of the activity of the hydrogen ion concentration of beach water sample (21) S(W)- Salinity (in ppt).**

In our study, all 21 variables of normalized data sets for the four different spatial regions (CL1-4) in our study were used for principal component analysis (PCA). The objective of this analysis was to see the compositional patterns between the beach water samples analyzed. The second objective was to identify the factors that influence each of the patterns. The input data (variable cases) used for the PCA was a 21 × 48 matrix. As suggested by Helena et al. (2000), the PCA was carried out using Minitab by a diagonization of the correlation matrix. Table 3 summarizes the PCA results obtained including the loadings (participations of the original variables in the new ones) and the eigenvalues of each principal component (PC). The amount of variance presented by each PC (also shown in the table) is dependent on its eigenvalues. Several literature exist that highlight criteria involved in the classification of principal components to be retained in order to understand the underlying data structure (Jackson, 2005;Helena et al., 2000). A scree plot was used to show change in the slope after the sixth eigenvalue. In numerical terms, eigenvalues above 1 was considered as the criteria for retention as PC. Consequently, PC1-PC6 were thus retained as they had eigenvalues higher than 1 and were responsible for 86.3% of the variance or information contained in the original data set. The absolute value of the loadings is an indicator of the participation of the parameter variables in the PCs. According to Helena et al. (2000), the actual sign is a reflection of the calculation algorithm used. In Table 3, the contribution reached by each variable to the considered principal component is presented. PC1 explained 33.4% of the variance and was contributed by ES, SW, D, S, DO and pH. PC2 explained 18.6% of the variance and was mainly contributed by EW, SAS, VSW, VSS, TCW. PC3 (12.9% of the variance) was contributed by VCS, VSS, TCS and FCW. PC4 (8.4% of the variance) is contributed by VSW, FCS, AW and T. The loadings (i.e. the contribution of each parameter towards the first and second principal components are also presented as a loading plot (Figure 4a). In the diagram, the loading of the first and second components are compared as they both are responsible for more than 50% of the variance. From the diagram it is observable that ES, TCPS, FCS, VSS, SAS all which are soil parameters weighed strongly in PC1 but negatively to the total variance observed in PC2. Observably for beach water, *Enterococci* (EW) appear to have an edge in that it weighed both strongly and positively in both first and second principal components unlike faecal coliforms (FCW) which weighed rather weakly in the first component and negatively in the second principal component.

**Table 3:** **PCA and FA loadings generated from principal component and factor analysis**

Variable	PC1	PC2	PC3	PC4	PC5	PC6		Factor1	Factor2	Factor3	Factor4	Factor5	Factor6	Communality
TPCW	−0.184	−0.069	−0.11	−0.153	−0.081	0.668		−0.371	−0.107	−0.085	0.108	0.077	0.842	0.882
TPCS	0.224	−0.094	−0.255	−0.309	−0.245	0.139		0.512	0.180	−0.636	−0.202	−0.173	0.274	0.845
EW	0.235	0.196	0.119	−0.179	−0.374	0.022		0.429	0.230	−0.027	−0.767	−0.123	−0.006	0.841
ES	0.304	−0.063	−0.144	−0.074	0.252	0.067		0.862	0.166	−0.172	0.165	−0.027	0.024	0.829
SW	0.315	0.030	−0.276	−0.042	0.157	0.104		0.935	−0.081	−0.264	0.019	−0.039	0.074	0.958
SAW	−0.031	0.278	0.021	0.015	−0.453	−0.178		−0.192	−0.270	0.007	−0.707	−0.117	−0.179	0.656
SAS	0.146	−0.353	0.051	0.236	−0.205	−0.200		0.114	0.459	−0.482	0.114	0.501	−0.367	0.855
VCW	−0.161	0.275	−0.278	0.145	0.228	−0.083		−0.111	−0.832	0.210	0.133	−0.208	−0.034	0.811
VCS	−0.03	−0.283	−0.332	−0.045	−0.116	−0.355		−0.128	−0.048	−0.769	0.352	−0.085	−0.234	0.796
VSW	−0.123	0.361	−0.198	0.319	−0.073	−0.048		−0.134	−0.868	0.218	−0.283	0.051	−0.111	0.915
VSS	0.103	−0.323	−0.31	−0.092	−0.277	0.124		0.161	0.164	−0.864	0.131	0.161	0.220	0.892
TCW	0.118	0.349	0.178	0.06	−0.337	0.153		0.213	−0.088	0.326	−0.835	0.077	0.036	0.864
TCS	0.144	−0.234	0.394	−0.044	−0.163	−0.188		0.034	0.846	−0.027	−0.127	0.197	−0.311	0.869
FCW	0.045	−0.128	0.467	−0.208	0.297	0.041		0.010	0.771	0.491	0.197	−0.079	0.004	0.881
FCS	0.221	−0.177	−0.031	0.389	−0.113	0.055		0.449	0.149	−0.282	−0.004	0.646	−0.196	0.760
AW	0.035	−0.141	0.171	0.443	−0.058	0.479		0.015	0.152	0.151	0.033	0.838	0.221	0.798
T	−0.196	−0.237	−0.022	0.429	0.01	−0.027		−0.526	−0.140	−0.110	0.433	0.547	−0.148	0.817
D	−0.35	−0.124	−0.035	−0.051	−0.159	0.032		−0.918	−0.141	−0.139	0.204	−0.041	0.199	0.965
S	−0.349	−0.12	−0.046	−0.047	−0.168	0.023		−0.917	−0.158	−0.154	0.196	−0.043	0.190	0.966
DO	−0.343	−0.032	0.203	−0.099	−0.112	−0.018		−0.950	0.047	0.213	0.053	−0.120	0.106	0.979
pH	−0.327	−0.14	−0.074	−0.241	0.01	−0.001		−0.795	−0.067	−0.141	0.365	−0.300	0.253	0.943
Eigenvalue	7.0098	3.909	2.7148	1.7736	1.4985	1.2161								
Proportion	0.334	0.186	0.129	0.084	0.071	0.058	Variance	6.0678	3.3253	2.7452	2.5779	1.9736	1.4317	18.1216
Cumulative	0.334	0.52	0.649	0.734	0.805	0.863	% Var	0.289	0.158	0.131	0.123	0.094	0.068	0.863

(1) TPCS-total heterotrophic plate count of beach sediment samples (2) EW- *Enterococci* concentration in seawater (3) ES- *Enterococci* concentration in beach sediment samples (4) SW- *Salmonella* typhi concentration in beach water samples (5) SAW- *Staphylococcus aureus* concentration in beach water samples (6) VCW- *Vibro cholerae* concentration in beach water samples (7) VCS- *Vibrio cholerae* concentration in beach sediment samples (8) VSW- *Vibrio* spp. concentration in beach water samples (9) VSS – *Vibrio* species concentration in beach sediment samples (10) TCW – Total coliform count of beach water samples (11) TCS- Total coliform count of beach sediment samples (12) FCW – Faecal coliform count of beach water samples (13) FCS- Faecal coliform count of beach sand samples (14) AW- *Aeromonas hydrophila* concentration in beach water samples (15) EW- *Enterococci* concentration in beach water samples (16) SAS- *Staphylococcus aureus* counts of beach sediment samples (17) T- Beach water temperature in 0C (18) D - Dissolved oxygen in % air saturation (19) DO - Dissolved oxygen in mg/L (20) pH- measure of the activity of the hydrogen ion concentration of beach water sample (21) S- Salinity (in ppt).

The values of PCA can be sanitized by means of verimax rotation (Helena et al. 2000). Using this approach, varivalues and VFs are produced in which original variables participate more clearly. Following the transformation, the significant VFs extracted using the same criteria as that for PCA yielded a spread of high variance among V1-V6. Table 3 shows the first six VFs spanning 80.3% of the variance as opposed to 86.3% as explained by the same number of PCs. The implicit combination of parameters in PC1 and PC2 is further broken up to clearly delineate parameters that weigh strongly for beach water and sediment quality monitoring. Using this approach, samples are ordered along the x-axis (Figure 4) in a similar but more compact way than was presented in Figure 4b. F1 and F2 are verimax-rotated PCs with a clearer picture of a cascade of traditional water quality parameters which weighted both strongly and positively among the two considered factors (using the same criteria used for the selection of PCs. Observably from Figure 4b, delineated parameters which weigh strongly for beach water were *enterococci* (EW) and faecal coliforms (FCW). The observation for beach water samples is consistent with available literature on the appropriateness of *enterococci* (EW) and faecal coliforms for surface water quality monitoring (Barrell et al. 2000). An approach to beach water quality monitoring may be that which at least focuses on the combination of EW and FCW for beach water quality monitoring.

On the other hand however, *enterococci* concentration in seawater (EW) weighed strongly in the first verifactor which was responsible for 28.9% of the total variance unlike faecal coliforms (FCW) which weighed strongly only in the second verifactor responsible for a lesser amount of variance (15.8%). Suggestively, *enterococci* concentration in seawater thus emerged as an indicator of choice for BQM in Malaysia. Previous studies have reported that the risk to health increases in proportion to the amount of faecal pollution as measured by indicator organisms. In line with the findings of our study that validate *enterococci* for use as indicator organism in BQM, a number of studies (Fleisher et al., 1993;Kay et al. 1992;USEPA, 2011) also attest that the bacterial indicator most strongly associated with risk to health, particularly for marine coastal beaches seems to be the *enterococci* count. Barrell et al. (2000) argues that although faecal coliforms were also independently associated with illness in some studies, the apparent superiority of *enterococci* as indicators of health risk in both drinking and bathing waters remains undisputed. The study however places a doubt on the willingness of relevant agencies in a period of 20 years to replace the traditional coliform standard with the apparently superior indicator, e*nterococci* as the primary routine microbiological test of water. Notwithstanding, in countries like Malaysia where there is currently no established beach water bacteriological monitoring program in place, a proactive approach for an envisioned BQM scheme will be an adoption of *Enterococci* as the choice parameter.

On the other hand, for beach sand delineated parameters; *Staphylococcus aureus* (SAS), faecal coliforms (FCS), *enterococci* (ES) and total plate count (TPCS) emerged with strong positive loadings significantly contributing to the variance following verimax rotation in factor analysis. This again highlights the importance of both to beach sand quality monitoring additionally, it is also suggested depending on the budget, an inclusion of SAS and TPCS as they generally may correlate with the levels of anthropogenic influence and subsequently contamination of coastal beach sand. Conditions that favor the persistence of autochthonous faecal indicator bacteria in coastal beach sand include increased protection from sunlight, buffered temperatures, nutrient availability, reduced osmotic stress, protection from predation by other microorganisms, a large surface area for biofilm development, and higher moisture and organic content from wave swash (Alm et al., 2003;Whitman et al. 2003; Heaney et al.,^2009^;Ishii et al. 2007;Beversdorf et al., 2007;WHO, 2003;Kinzelman et al. 2004;Yamahara et al., 2009). In the light of these factors, studies have thus shown that microbial contamination is higher in sand than in adjacent waters, as the sand behaves as a passive harbour for cumulative pollution (Oliveira & Mendes, 1991,1992;Oshiro & Fujioka, 1995). This could subsequently become the reason for periodically high levels of bacteria in the adjacent water. Moreover, a hard and bind rule is not easy to achieve when making a case for guideline values of faecal indicator organisms in sand water because sand contamination is highly variable over short distances, making interpretation of results difficult (Figueras et al., 1992;Oshiro & Fujioka, 1995).

Also, little is known about inter-linkages between sand contact activities and health effects and only a few studies have attempted relating sand contact activities to increased risk of illness among beachgoers (Marino et al., 1995;Bonilla et al. 2007, Heaney et al., 2009). Nonetheless, the importance of beach sand in relation to recreational health risks may be well underestimated particularly for tropical climates. WHO (2003) argues that the presence of indicator bacteria in beach sand and the relationship between their counts in beach sand and their counts in adjacent waters have comprised a significant area of research, with apparently contradictory results. The report also argued that the capacity of pathogens in beach sand to infect beach users remains undemonstrated, and the real extent of their threat to public health is unknown and that there is no evidence to support the establishment of a guideline value for index organisms or pathogenic microorganisms in beach sand. However, the studies whose findings formed the basis for these conclusions were largely conducted on temperate beaches. For example, all the citations referred to {Conseil Supérieur d’Hygiène Publique de France, (1990) (France), Signorile et al. (1992), Bonadonna et al. (2002) (Italy), Figueras et al. (1992), Ghinsberg et al. (1994) (Spain), Papadakis et al. (1997), Borrego et al. (1991) (Greece)} are locations where daily ambient temperatures are by far lower than what obtains in the tropics. Conclusions drawn on occurrences or observation in temperate waters of the world may not present adequate representations of what obtains generally on the globe. Understandably however, studies that have focused on beach sand quality in tropical beaches are few. Hopefully, more studies will emerge in the future particularly in tropical climates that will explore this area of research. It is desired that such studies attempt to provide epidemiological evidence for health risks from exposure to sandy beaches while exploring possible dose–response relationships that associate the microbial quality of beach sand with infectious diseases.

Our findings provide meaningful evidence particularly as it relates to the correlation of *Enterococci* with pathogens and other non-microbial parameters. It also provides empirical data to validate the applicability of the *enterococci* indicator paradigm for BQM in Malaysia. The current study thus provides policy decision makers evidenced based approach to parameter streamlining for optimized beach sampling and sustainable bacteriological quality monitoring. *Enterococci* counts in beach water samples (EW) emerged with strong loadings and correlations in our analysis, thus signifying its appropriateness for use as indicator in BQM in the selected multi-beaches. The application of our findings could be up-scaled to the national level by relevant agencies charged with the mandate of coastal water management. A practical limitation however in its application for beach water quality analysis and beach profiling is the unavailability of similar studies in the country that employ this approach. This makes comparison difficult. To the best of our knowledge, this is the first study that uses a combination of multi-variate and multi-parameter approach for the validation of *Enterococci* as indicator organism for bacteriological quality monitoring of recreational beach water quality in Malaysia. Hopefully studies will emerge in the future with a view to providing policy decision makers validated empirical data that correlates *Enterococci* with even a larger pool of pathogens and non-microbial parameters in recreational beaches in Malaysia.

## Conclusion

Different multivariate statistical techniques were used to evaluate spatial variations of beach water quality and to validate *enterococci* as an appropriate indicator organism for BQM in Malaysia. Correlation analysis showed that *enterococci* correlated to more number of beach water and beach sand quality parameter tested in this study. A simple regression model generated in this study was able to predict with a maximum predictive success of 69.64% using just a combination of 5 parameters. Hierarchical clustering grouped 41 sampling locations into four clusters of similar water quality characteristics. The factor analysis/principle component analysis resulted in significant data reduction and assisted in extracting the water quality parameter that weighed strongly in the observed variations in beach river water quality at the different sampled beaches. Although delineated parameters with significant weighing for beach water were *enterococci* (EW) and faecal coliforms (FCW), *enterococci* concentration in seawater presented stronger loadings and suggestively emerged as an appropriate indicator of choice for BQM in Malaysia. This study also illustrates the usefulness of multivariate statistical techniques for the validation of the use of *enterococci* as indicator organism in BQM.

## Methods

### Study area

**Figure 5:**
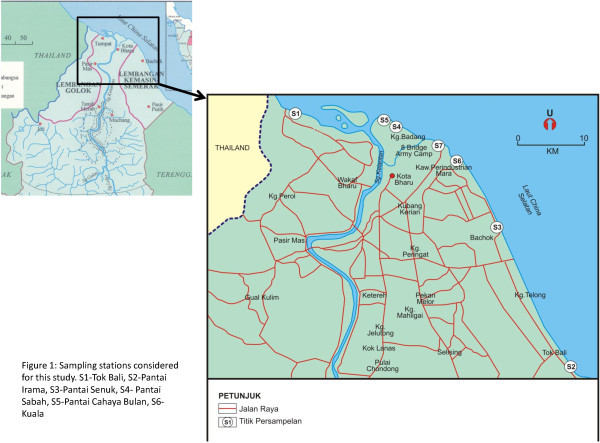
**Sampling stations considered for this study.** S1-Pantai Sri Tujuh (SRI7), S2-Tok Bali, S3-Pantai Irama, S4- Pantai Cahaya Bulan (PCB), S5-Kuala Besar, S6-Pantai Senuk, S7- Pantai Sabak.

The beaches considered for sampling during this study are locations selected as part of a nation-wide pathogen monitoring program under the auspices of the School of Bioscience and Biotechnology, Faculty of Science and Technology, Universiti Kebangsaan Malaysia. The samples were taken at the public beaches at Kelantan (Figure 5). Pantai Seri Tujuh lies on the border of Thailand and Kelantan at Kampung Tujuh in Turnpat, about 7 km from Kota Bharu. The coastline fringes the South-China Sea en-route to Pantai Seri Tujuh. Tok Bali Beach is located near the mouth of Semerak Canal. Irama Beach (Beach of Melody) is a wide and long sandy beach situated in Bachok district. It is about 25 km south of Kota Bharu and faces the South China Sea with. This beach has over the past decade suffered from coastal erosion. Pantai Cahaya Bulan is located some 10 km from Kota Bharu town. Spanning about 1.2 kilometers, this beach is famously known as the landing area for the Japanese-British war in the formative years of Malaysia. Two important fishing villages in the East Coast of Malaysia are Sabak Beach (approx. 14 km from Kota Bharu) and Kuala Besar (15 km from Kota Bharu).

### Sample collection

Sterile glass bottles (1,000 ml) were used to collect water samples in duplicates, while sterile disposable containers (500 ml) were used to collect sediments. Samples were stored on ice until analyzed, usually within 4 h after collection.

### Analysis of physicochemical parameters

Physico-chemical parameters were measured on site. Samples were stirred gently and stable readings were recorded as described by Sangiorgio et al. (2010) using YSI 556 hand-held multi-probe that simultaneously measures Dissolved Oxygen (% and g/L), pH, Conductivity (mS/cm), TDS (g/L), Salinity (ppt) and Temperature (°C). Readings were recorded in triplicates.

### Enumeration of *Enterococci* and other pathogens

*Enterococci* were recovered from seawater using membrane filtration method using Slanetz and Bartley (S + B) culture media as previously described (Dada et al., 2013). Counts were recorded as cfu/ml. Isolates that were catalase negative, able to grow in 6.5% NaCl and to hydrolyze esculin in the presence of 40% bile salts were considered as presumptive *Enterococci*. *Vibrio* was enumerated using Thiosulfate Citrate Bile salt Sucrose (TCBS) agar, a growth medium generally used for their detection as described (Cañigral et al., 2010). *Aeromonas hydrophila* was recovered using modified Rimler Shotts agar. The media consisted of L-lysine (5 g/L), L-ornithine (6.5 g/L), maltose (7.5 g/L), sodium thiosulphate (6.8 g/L), L-cysteine (0.3 g/L), ferric ammonium citrate (0.8 g/L), bile salt (5 g/L), yeast extract (3 g/L), bacto agar (13 g/L), bromothymol blue (0.03 g/L) all prepared in a litre of distilled water. Plates were incubated at 37°C for 24-48 h.

For *Staphylococcus aureus,* manitol salt agar at 35°C ± 2°C for 24 h was used (Goodwin et al. 2012). After incubation, yellow colonies were counted as presumptive *S. aureus*. For confirmation, from each sample lot representative colonies of *S. aureus* were chosen and subjected to the coagulase test. The total viable bacterial and coliform populations were enumerated in seawater and sediment samples by spread plate method using nutrient agar medium (Himedia) with 2.5% NaCl supplementation and chromogenic coliform agar respectively. For faecal coliform counts, the same agar was used as for total coliform except that plates were incubated at elevated temperatures of 44.5°C (xAPHA, 1999). Aliquots of seawater samples were serially diluted with sterile saline and 100 ul of samples were spread on nutrient agar medium. For sand samples, a different approach was used. Each sediment samples were weighed, added in to sterile saline (1:100) and thoroughly shaken to ensure that all attached microbes are disentangled from the sediment. Subsequently, the procedure used for enumeration of seawater was followed for total viable count using the supernatant from the sand-saline suspension. Experiments were performed in triplicates and numbers of bacterial colonies were expressed in logCFU/g of beach sand and logCFU/100 ml of seawater. Specific media (Salmonella-Shigella agar) was used for identification of *Salmonella typhi* (Wells and Butterfield, 1997). All the plates were incubated in triplicates at 37°C for 24–48 h.

### Descriptive statistical analyses

The purpose of descriptive statistics was to explore (i) general frequencies of indicator organism and pathogen count at the study sites and ii) to present a geometric mean-based correlation analyses to explore relationships between *Enterococci* and other microbial and non-microbial parameters measured in the study. A two-way ANOVA was done with each row in the tables generated representing a different time point such that matched values are stacked together into a sub-column. The experimental design selected for the analysis was such that the factor that defined the columns was the parameters considered and the factor that defined the rows was the respective beach studied. Multiple comparisons were also conducted involving comparisons within each column, comparing rows (simple effects within columns). Using this approach, for each column, comparisons of cell mean was made with every other cell mean on each column. Turkey’s test was used to correct for the multiple comparisons and to compute confidence intervals (CIs) and significance. Column-wise significance and confidence levels were taken as 0.05 and 95% respectively. All generated data were fed into a grouped data format and column statistics involving correlations made using the Pearson’s Correlation rank.

### Regression analysis

Following the test for associations between recorded parameters (considered in pairs) using the Pearson Correlation rank analysis, multiple linear regression was also conducted comparing one dependent and multiple independent variables. Using a more or less descriptive approach, the purpose of the multiple linear regressions was to highlight a best fit model that could function in predicting *enterococci* concentrations as it relates to other parameters in beach water and sand samples. As described by Goodwin et al. (2012), model selection was based on p-values for the coefficient (apha =0.05). Non-significant terms were sequentially removed from the model using stepwise method. The formula used for regression analysis in our study was adapted after Camdevyren et al. (2005) and is stated as:2

Where a is the constant term, b^k^ is the regression coefficient of values of parameter s^k^, e is the error term of the model. In this study, *t*-test was used in testing the regression coefficients. Analysis of variance (ANOVA) was also conducted for the model with p taken as significant at values less than 0.05.

### Multivariate statistical methods

As suggested by Shrestha and Kazama (2007), the Kolmogorov-Smirnov (K-S) statistics were used for the purpose of confirming the goodness-of-fit of the data to log normal distribution at a 95% confidence level. Four major multivariate statistical methods were used in the multi-beach study: cluster analysis (CA), principal component analysis (PCA), factor analysis (FA) and discriminate analysis (DA). The purpose of the application of multivariate statistical analysis to our data was primarily to help in the interpretation of complex data matrices and to ultimately present a more meaningful understanding of water quality of the studied beaches. This unarguably offers policy decision makers in their efforts aimed at sustainable management of coastal beach water resources (Adams et al., 2001, Alberto et al., 2001;Simeonov et al., 2003;Singh et al., 2004,2005). Data generated for all the parameters were z-scale transformed before used in line with the suggestions of Liu et al. (2003) and Simeonov et al. (2003). This transformation was necessary to prevent misclassifications arising from the different orders of magnitude of both numerical values and variance of the parameters analyzed. Softwares used in the analysis are Minitab 16, SPSS 20 and Graph Prism.

### Cluster analysis

The major reason why this analysis was done was to assemble the sampled sites based on the characteristics they possess. Cluster analyses is an unsupervised pattern recognition method that divides a large group of cases into smaller groups or clusters of relatively similar cases that are dissimilar to other groups (Zhou et al., 2007). One of the most common approach used in CA is Hierarchical agglomerative, a method typified by its provision of similarity relationships between any one sample and the entire data set. Usually, it begins with each case in a separate cluster and joins the clusters together step by step until only one cluster remains (Lattin et al., 2003;McKenna, 2003). The output is usually illustrated by a dendrogram which presents the groups and their proximity pictorially alongside with a reduction in dimensionality of the initial data fed into the analyses (McKenna, 2003). During the analyses, Euclidean distances taken as a measure of similarity using the Ward’s approach was adopted for the hierarchical agglomerative CA.

### Principal component analysis

The purpose of PCA in the study was to transform the original variables into new, uncorrelated variables (axes), called the principal components, which are linear combinations of the original variables. Using this analysis, a more purposeful approach was achieved for detailing the source of variation in the data (Sarbu and Pop, 2005). Other important goals achievable through the principal component analyses are substantial data reduction while still retaining original information (Helena et al., 2000). The PCA is described by the formula:3

where z is the component score, a is the component loading, x the measured value of variable, i is the component number, j the sample number and m the total number of variables.

### Factor analyses

Factor analysis was done after Principal Component Analyses. Factor analyses (FA) is particularly applicable in water quality monitoring where series of approaches consisting of varied mix of indicator organism and pathogen detection approaches are used. FAs help to reduce the contribution of less significant variables to simplify even more of the data structure coming from PCA. To run this analysis, the axis defined by PCA was carefully rotated with the subsequent formation of new variables termed as varifactors (VF) (Vega et al., 1998;Helena et al., 2000). In other words, the contribution of variables with minor significance is further decreased as only significant PCs extracted from PCA are verimax rotated to generate VFs in the FA. FA is based on the premise that a small number of factors will usually account for approximately the same amount of information as do the much larger set of original observations (Brumelis et al., 2000;Singh et al., 2004,2005;Love et al., 2004;Abdul-Wahab et al., 2005). Factor analysis is given by the formula:4

where z is the measured variable, a is the factor loading, f is the factor score, e is the residual term accounting for errors or other source of variation, i the sample number and m the total number of factors.
